# Eccentric Countermovement Jump Mechanics Are Associated with Bone Properties in Physically Inactive Early Postmenopausal Women

**DOI:** 10.3390/sports14090373

**Published:** 2026-09-01

**Authors:** Salvatore Pinelli, Gonzalo Reverte-Pagola, Sergio Tejero, Laura Bragonzoni, Borja Sañudo

**Affiliations:** 1 Department for Life Quality Studies, University of Bologna, Campus Rimini, Corso D’Augusto 237, 47921 Rimini, Italy; salvatore.pinelli2@unibo.it (S.P.); laura.bragonzoni4@unibo.it (L.B.); 2 Department of Physical Education and Sport, University of Seville, C/Pirotecnia, s/n, 41013 Seville, Spain; sertejgar@us.es (S.T.); bsancor@us.es (B.S.); 3 School of Humanities, Education and Sport, Universidad CEU Fernando III, CEU Universities, Glorieta Ángel Herrera Oria, s/n, 41930 Bormujos, Spain; 4 Grupo de Investigación Sport, Performance, Health, Exercise, Research (SPHERE), School of Humanities, Education and Sport, Universidad CEU Fernando III, CEU Universities, 41930 Bormujos, Spain; 5 Foot and Ankle Unit, Orthopaedic Department, Department of Surgery, University Hospital Virgen del Rocío, University of Seville, Avda Doctor Fedriani, s/n, 41013 Seville, Spain; 6 School of Clinical Therapies, College of Medicine & Health, University College Cork, T12 AK54 Cork, Ireland

**Keywords:** jump biomechanics, bone properties, bone microarchitecture, stretch-shortening cycle, menopause, exercise testing

## Abstract

Countermovement jump (CMJ) assessment is widely used to evaluate lower-body neuromuscular performance, but its potential relationship with bone health remains insufficiently understood. This study aimed to determine whether eccentric-phase force–time characteristics during the CMJ are associated with proximal femur bone density, mass, and structural properties in postmenopausal women. One hundred and ten physically inactive women within 10 years after menopause underwent bone and jump assessments. Areal bone mineral density and bone mineral content were measured using dual-energy X-ray absorptiometry, and three-dimensional cortical and trabecular proximal femur parameters were estimated from dual-energy X-ray absorptiometry images. Participants performed maximal countermovement jumps on a force platform. Seventeen eccentric-phase force–time variables were extracted and reduced using principal component analysis. Multivariable linear regression models adjusted for age, body mass, height, waist circumference, and hip circumference examined associations between eccentric mechanical profiles and bone outcomes. Four principal components explained 90.5% of the variance in eccentric jump mechanics. Regression models explained between 11% and 41% of the variance across bone outcomes, with the strongest models observed for trabecular bone mineral content at the femoral neck (R^2^ = 0.41), bone mineral content at the femoral shaft (R^2^ = 0.40), and total trabecular volume (R^2^ = 0.39). The component reflecting greater countermovement depth and longer eccentric duration showed the most consistent contribution across models, whereas the force–power component contributed to selected trabecular outcomes. These findings suggest that the combined eccentric mechanical profile of the CMJ is associated with proximal femur bone properties in physically inactive women in early postmenopause and may help inform the biomechanical assessment of bone health and the development of bone-targeted exercise interventions in this population.

## 1. Introduction

Postmenopausal bone fragility is driven not only by the loss of bone mass but also by deterioration of bone microarchitecture. Following menopause, estrogen deficiency accelerates remodeling imbalance, increases bone turnover, and promotes rapid losses in bone mineral density (BMD), particularly during the early postmenopausal years [1]. In parallel, trabecular bone undergoes thinning, increased separation, loss of connectivity, and greater structural heterogeneity, changes that compromise mechanical competence beyond what can be captured by areal BMD alone [2,3]. These deficits are especially relevant at trabecular-rich skeletal sites and help explain why fragility fractures remain a major public health burden in postmenopausal women [4]. Recent reviews emphasize that osteoporosis and fragility fractures continue to impose substantial consequences for pain, disability, loss of independence, and survival, and that approximately one in three women over 50 years of age will sustain an osteoporotic fracture during their remaining lifetime [5].

The biological rationale for exercise-based prevention is grounded in Frost’s mechanostat concept, now interpreted through contemporary osteocyte biology [6]. In its modern form, the mechanostat proposes that bone mass and structure adapt to habitual mechanical usage, whereas insufficient loading promotes resorption and appropriately intense loading promotes modeling and remodeling responses [6]. Current evidence identifies osteocytes as the primary mechanosensors regulating this process, promoting osteoblast differentiation and bone formation [7]. Importantly, bone adaptation is not governed by load exposure per se, but by specific features of the loading signal, including magnitude, rate, frequency, and spatial distribution. High-rate, dynamic, and heterogeneous loading appears especially osteogenic because it enhances local strain gradients and fluid flow within the lacuno-canalicular network, thereby amplifying mechano-transduction [7]. Current mechanobiological models further propose that the osteogenic response reflects the integrated mechanical loading history experienced by bone rather than isolated peak loading characteristics. Consequently, temporal aspects of loading may contribute to the overall mechanical stimulus by modifying the force–time history experienced by bone tissue, although direct evidence specifically linking these temporal characteristics to skeletal adaptation remains limited [8,9,10,11].

These principles are highly relevant to jumping exercise. Meta-analytic evidence indicates that, in postmenopausal women, higher-intensity bone-targeted exercise elicits greater lumbar spine BMD benefits than low- or moderate-intensity loading, reinforcing the importance of load magnitude and intensity as key determinants of skeletal adaptation [12]. Likewise, high-intensity resistance and impact training can be both efficacious and well tolerated in appropriately selected postmenopausal women with low bone mass [5]. Mechanically, jumping and related high-impact tasks are attractive because they impose large forces over short time intervals, thereby maximizing loading rate, a recognized surrogate of strain rate. However, the conventional view that their osteogenic effect is explained mainly by external impact is now being challenged [13,14]. Recent work shows that countermovement jumping, running, and hopping differ markedly in joint loading profiles, and that ground reaction forces alone may not adequately represent skeletal loading at specific joints because muscle activation and joint contact forces make major contributions to the internal load experienced by bone [13]. Similarly, a recent critical review concluded that external ground reaction force metrics are only weakly related to tibial bone load in dynamic tasks, further underscoring that internal musculoskeletal loading cannot be inferred from impact measures alone [14].

Beyond its role as an osteogenic exercise, the countermovement jump (CMJ) has recently emerged as a potential biomechanical marker of skeletal health. Cross-sectional studies in physically inactive postmenopausal women have demonstrated that individual CMJ-derived variables, including peak landing force and peak force, are positively associated with proximal femur BMD, with force-based metrics explaining up to 32% of the variance in regional hip BMD after adjustment for anthropometric covariates [15]. Moreover, peak force has recently been shown to discriminate women with low total hip BMD with moderate diagnostic accuracy (AUC = 0.78), suggesting that force platform assessment during a CMJ may have potential as a preliminary screening tool for skeletal health [16]. However, these studies have primarily focused on isolated CMJ-derived variables, such as jump height, peak force, peak power, or landing force, without considering the multidimensional mechanical organization of the eccentric phase. Consequently, it remains unknown whether distinct eccentric loading profiles, integrating force, power, velocity, impulse, movement amplitude, and temporal characteristics, are differentially associated with conventional DXA measures and three-dimensional cortical and trabecular bone properties. Addressing this gap may provide a more comprehensive understanding of the biomechanical characteristics of the CMJ that are most relevant to skeletal adaptation. Whether these characteristics have implications for exercise design remains to be established in longitudinal and intervention studies.

This question is particularly relevant in postmenopausal populations, in whom repeated high-impact landings may be poorly tolerated or avoided because of frailty, fear of injury, or pelvic floor symptoms [17]. Contemporary evidence indicates that urinary incontinence is a meaningful barrier to physical activity in women, and that high-impact exercise is associated with greater rates of stress urinary incontinence [18]. Accordingly, identifying the eccentric mechanical characteristics that are most strongly associated with bone properties may help generate hypotheses regarding movement strategies that warrant evaluation in future exercise intervention studies. Against this background, the stretch-shortening cycle (SSC) embedded within the CMJ may be clinically and biologically important. The rapid eccentric-to-concentric transition increases force output and rate of force development before take-off, and may therefore generate a substantial osteogenic signal even before landing [12,19]. This is consistent with experimental evidence showing that CMJs can provide a greater putative osteogenic stimulus than other commonly prescribed osteoporosis-prevention exercises [18], and with the broader mechanobiological literature showing that dynamic, high-rate loading activates osteocyte-driven anabolic pathways [7]. However, beyond loading rate, the eccentric phase of the SSC also encompasses modifiable temporal and coordinative features (e.g., depth and duration) that influence the force–time characteristics of movement and therefore may modify the integrated internal mechanical loading experienced by bone. Although direct experimental evidence specifically linking these temporal characteristics to skeletal adaptation remains limited, their potential contribution to the overall mechanical stimulus provides a biomechanical rationale for their investigation. At the trabecular level, such loading is especially relevant because dynamic and spatially heterogeneous stimuli are thought to favor targeted adaptation of the trabecular network; indeed, microarchitecture-based assessments improve fracture prediction beyond dual-energy X-ray absorptiometry (DXA) alone in postmenopausal women, highlighting the functional importance of trabecular organization for skeletal fragility [2]. Nevertheless, recent studies also show that exercise effects on trabecular microarchitecture in older adults remain incompletely characterized, largely because of the small number of studies and the limited range of loading modalities tested [20]. Accordingly, examining not only the magnitude but also the temporal organization of SSC-related mechanics during a CMJ may help determine whether distinct eccentric loading profiles are differentially associated with DXA- and 3D-DXA-derived bone properties in physically inactive women during early postmenopause, differentially associated with bone health in postmenopausal women. Because the eccentric phase of the CMJ reflects a complex interaction between movement strategy, force production, velocity, power generation, impulse, and temporal coordination, it cannot be adequately characterized using a single force platform variable. Instead, these complementary descriptors collectively define the mechanical loading profile experienced during the stretch-shortening cycle and provide a more comprehensive representation of the internal mechanical stimulus potentially relevant to bone adaptation. Therefore, the aim of the present study was to determine whether distinct biomechanical profiles of the eccentric phase of the SSC, identified through principal component analysis of CMJ force–time variables, were associated with BMD and cortical and trabecular bone characteristics in physically inactive women within the first 10 years after menopause.

We hypothesized that, in this specific population, distinct eccentric-phase CMJ mechanical profiles would be differentially associated with proximal femur BMD, BMC, and 3D-DXA-derived cortical and trabecular parameters, with profiles characterized by greater force–power output and/or longer countermovement duration showing stronger associations with these bone properties.

## 2. Materials and Methods

### 2.1. Participants

A total of 120 postmenopausal women (mean age 56.9 ± 3.8 years; range 46–68 years) were recruited for this study. Postmenopausal status was confirmed by a gynecologist based on the absence of menstruation for at least 12 consecutive months. Only women were included because the research question specifically addressed early postmenopausal bone health, a female-specific physiological condition characterized by menopause-related changes in bone metabolism and fracture risk. The protocol was approved by the Andalusian Biomedical Research Ethics Committee (approval code: 0486-N-22) and registered at ClinicalTrials.gov (NCT06741956; 15 December 2024). Participant recruitment and screening followed a previously described protocol [21]. In brief, participants were recruited through the gynecology units of Virgen del Rocío University Hospital (Seville, Spain).

Eligible participants were physically inactive postmenopausal women aged >40 years, within 10 years since menopause, and reporting <150 min/week of moderate-to-vigorous physical activity over the previous 6 months. Women within the first 10 years after menopause were specifically selected because this period represents the phase of most rapid estrogen deficiency-related bone loss, characterized by accelerated bone remodeling and marked deterioration of trabecular and cortical bone. Restricting recruitment to this early postmenopausal window also reduced biological heterogeneity associated with later age-related skeletal deterioration while focusing on the period in which preventive strategies aimed at preserving bone health are considered to have the greatest potential clinical impact [22]. Similarly, only physically inactive women were included to minimize the confounding influence of long-term exercise-induced adaptations on both bone properties and CMJ biomechanics, thereby improving the internal validity of the biomechanical analyses. Exclusion criteria included surgically induced menopause, ongoing cancer treatment, body mass index < 18 kg/m^2^, excessive alcohol consumption (>14 drinks/week), current smoking, recent hormone replacement therapy (≤3 months), fractures within the previous 6 months, mobility limitations requiring assistance, or participation in structured exercise programs during the previous 6 months. All participants provided written informed consent prior to inclusion. The study was designed to recruit a representative sample of physically inactive early postmenopausal women rather than a disease-free cohort. Therefore, women with osteopenia were eligible for inclusion provided they met all eligibility criteria and had no contraindications to participation. During the baseline assessment, detailed information regarding medical history, medications, lifestyle factors, fracture risk factors, and potential secondary causes of bone loss was collected according to the study protocol. Common age-related comorbidities were not considered exclusion criteria unless they interfered with safe participation or met one of the predefined exclusion criteria.

A priori sample size was calculated using G*Power software (version 3.1.9.7) for multiple linear regression analysis via F-test family, testing the R^2^ increase (fixed model, full general linear model). The model incorporated nine predictors (four principal components from PCA together with five prespecified covariates: age, body mass, height, waist circumference, and hip circumference), with parameters set to medium effect size f^2^ = 0.15, two-tailed α = 0.05, and statistical power of 0.80 (1 − β). This yielded a required minimum sample of *n* = 110 participants. To compensate for an expected 10% data attrition due to motion capture errors, final recruitment targeted *n* = 120 individuals. All 120 participants completed the assessment protocol. Participants without at least one technically valid CMJ trial after quality-control procedures were excluded from the biomechanical analyses, resulting in a final analytical sample of 110 women.

### 2.2. Bone Assessment

Participants underwent DXA using a Lunar Prodigy Advance system (GE Healthcare, Chicago, IL, USA). BMD (g/cm^2^) and bone mineral content (BMC; g) were assessed at the right proximal femur, including the femoral neck, greater trochanter, and femoral shaft. The scanner was calibrated daily using a manufacturer-provided phantom to ensure measurement stability. A site-specific least significant change value was not available because repeated repositioned DXA scans were not performed in this cohort. DXA-derived measures have demonstrated excellent reliability, with reported intraclass correlation coefficients exceeding 0.99 [23]. In addition to standard DXA-derived measures, three-dimensional cortical and trabecular bone parameters at the proximal femur were estimated using 3D-Shaper software, version 2.14 (Galgo Medical, Spain). This technique reconstructs subject-specific three-dimensional bone models from two-dimensional DXA images through a statistical modeling approach, enabling the assessment of volumetric and structural properties. Trabecular parameters included trabecular volumetric BMD (vBMD; mg/cm^3^), trabecular BMC (BMC; g), and trabecular volume (cm^3^), reflecting the density, quantity, and spatial distribution of the trabecular network. Cortical-related outputs were derived from the integral compartment and included integral vBMD (mg/cm^3^) as a surrogate of combined cortical and trabecular density, as well as geometric measures such as cross-sectional area (cm^2^), which provide indirect information on cortical bone distribution and structural strength. These parameters have been shown to correlate with three-dimensional imaging modalities and provide improved insight into bone strength beyond areal DXA measurements [24,25].

### 2.3. Countermovement Jump Assessment

Before jump testing, participants completed a standardized warm-up consisting of approximately 5 min of self-paced walking followed by dynamic lower-limb mobility exercises targeting the hips, knees, and ankles. Subsequently, participants performed three to five submaximal CMJs under investigator supervision to familiarize themselves with the testing procedure, force platform, and movement technique. Maximal testing commenced only after participants demonstrated that they could perform the movement safely and correctly. Because the study population consisted of physically inactive postmenopausal women, the familiarization procedure was primarily intended to ensure participant safety, increase confidence, and minimize potential learning effects rather than optimize performance.

During testing, participants were instructed to “jump as high as possible” while keeping their hands on their hips throughout the movement. They were allowed to use a self-selected countermovement depth and comfortable movement strategy, without specific instructions regarding countermovement depth or descent velocity. This approach was intentionally adopted because the study involved physically inactive postmenopausal women, for whom participant safety and natural movement execution were prioritized while still encouraging maximal vertical jump performance. Standardized verbal encouragement was provided during every trial. During the assessment participants stood motionless on the platform with hands on hips for ≥2 s to determine body mass, and subsequently performed maximal CMJs upon a standardized verbal cue. Each participant completed three maximal trials separated by 60 s of rest, with consistent verbal encouragement provided throughout.

A total of 360 CMJ trials were initially recorded from 120 participants. Of these, 56 trials were discarded due to acquisition errors, such as loss of foot contact with the force plate or the occurrence of double bounces during the countermovement phase, resulting in 304 valid trials. Ten participants did not complete at least one technically valid CMJ trial after quality-control procedures and were therefore excluded from the biomechanical analyses. For the remaining 110 participants, the valid trial achieving the greatest jump height was retained for subsequent analyses. For participants with more than one valid trial, the CMJ achieving the greatest jump height was selected for analysis. This approach was adopted because the objective of the study was to characterize each participant’s maximal eccentric mechanical capacity rather than average performance across repeated attempts. The highest valid trial is considered the most representative of the maximal neuromuscular output that an individual can achieve under standardized conditions while reducing the influence of submaximal efforts, pacing strategies, or hesitation, which may be particularly relevant in physically inactive postmenopausal women. Because the study sought to investigate the relationship between maximal mechanical loading and bone properties, the best valid trial was considered the most appropriate representation of the osteogenic stimulus generated during the CMJ.

### 2.4. Data Processing

**Figure 1:**
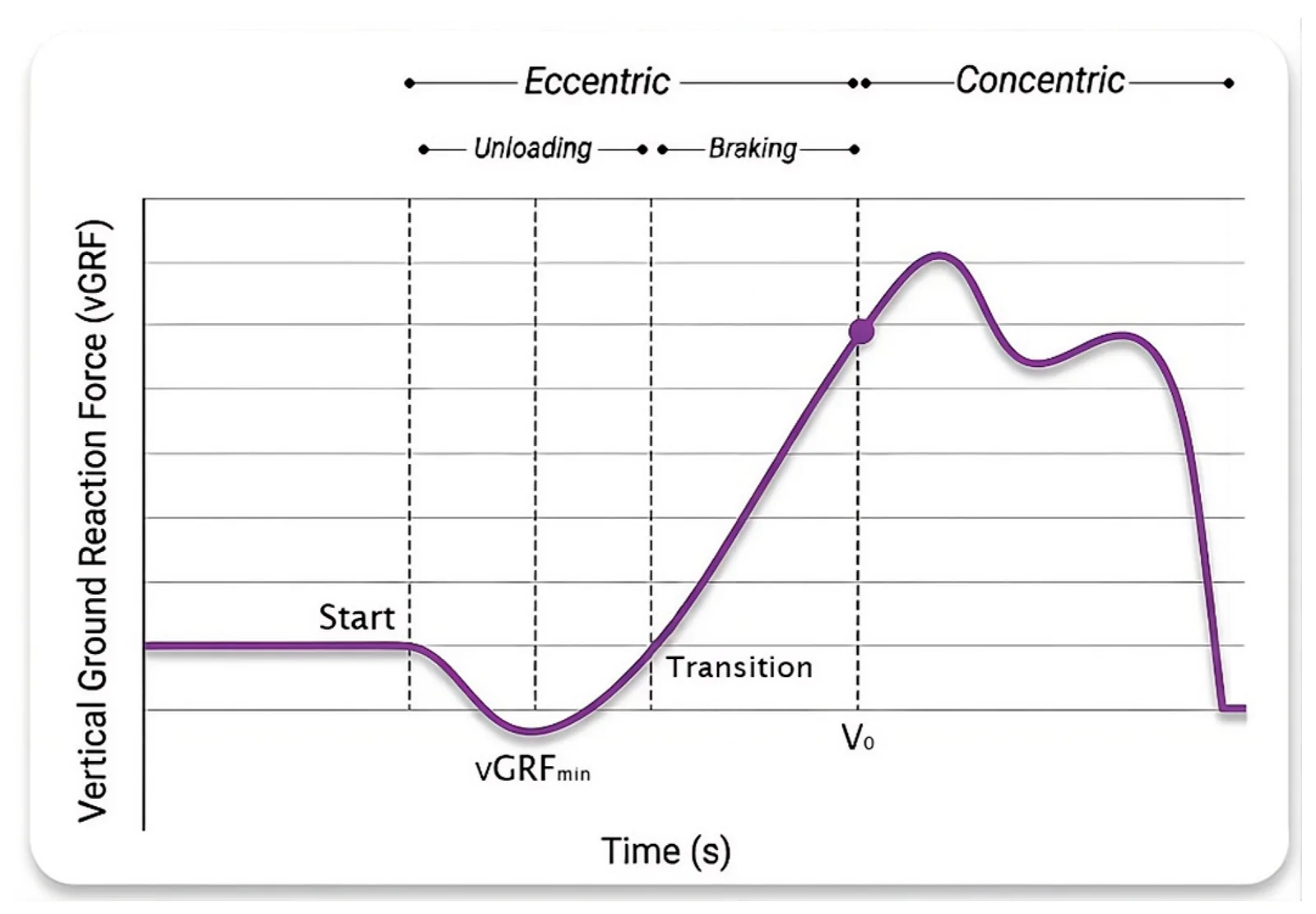
Representative vertical ground reaction force (vGRF) curve from a countermovement jump (CMJ) performed on a force plate. Vertical lines denote key temporal landmarks: START (movement onset), vGRF_min_, TRANSITION (onset of braking phase), and V_0_ (marking the end of the eccentric phase and start of the concentric phase).

Vertical ground reaction force (vGRF) data were recorded using a previously calibrated force plate (Kistler, Switzerland) at a sampling frequency of 1000 Hz. The vGRF data obtained was processed using custom-made software in Python (Python Software Foundation; Python Language Reference, version 3.13.5). vGRF data were low-pass filtered using a second order Savitzky–Golay filter with a window size determined as described by Vivó-Truyols and Schoenmakers [26]. Body weight (BW) was determined during the initial motionless standing period. Net vGRF was computed by subtracting BW from absolute vGRF, and centre of mass (COM) acceleration was derived by dividing net vGRF by participant body mass. COM velocity and displacement were then obtained via numerical integration and double integration of acceleration data, respectively [27,28]. All force-derived variables were normalized to BW. Key temporal landmarks were identified as follows (Figure 1): movement onset (START) occurred when vGRF dropped 2.5% below steady-state BW; vGRFmin marked the global vGRF minimum during the eccentric phase; TRANSITION was defined as the point when vGRF rose back to BW level post-vGRFmin (end of unweighting and onset of braking); and V_0_ (zero-velocity crossing) represented the velocity sign-change instant, signaling the end of the eccentric phase and start of the concentric phase, typically near but distinct from peak force [29].

Seventeen biomechanical variables were selected to comprehensively characterize the eccentric phase of the CMJ (Table 1). Rather than representing isolated performance outcomes, these variables were chosen to describe the principal mechanical domains of eccentric stretch-shortening cycle function, including movement strategy (countermovement depth and temporal characteristics), velocity, force production, power generation, and impulse development. Collectively, these variables provide a multidimensional representation of the eccentric loading profile, allowing the mechanical strategy adopted during the CMJ to be characterized. Because these descriptors are highly interrelated, principal component analysis was subsequently used to identify the dominant underlying mechanical constructs while reducing dimensionality. Variables were calculated according to established force platform methodologies [30,31]. These parameters were categorized into kinematic variables (movement timing and velocity metrics) and kinetic variables (force, power, and impulse metrics), as detailed below.

### 2.5. Statistical Analysis

Within-session reliability for the 110 subjects was determined from the original testing session by means of repeated-measures ANOVA, yielding Intraclass Correlation Coefficients (ICC, model A,1). The ICC(A,1) model was selected for all measured variables, as it accounts for systematic bias between repeated measurements. To characterize absolute reliability, Coefficients of Variation (CV%) and the Standard Error of Measurement (SEM) were also computed, providing an index of within-trial variability expressed as a percentage. The within-subject coefficient of variation (CV%) was calculated for each participant as the ratio between the standard deviation and the mean of their repeated trials, and the reported CV% represents the average of these individual coefficients across all participants.

Principal component analysis (PCA) was used to generate a new set of linearly uncorrelated variables that summarize the original 17 dependent biomechanical variables [32,33]. The analysis was performed in Python (Python Software Foundation; Python Language Reference, version 3.13.5) after standardizing all variables to account for differences in scale. PCA, implemented via singular value decomposition, transforms the original variables into orthogonal components (PCs) that capture their shared variance. These components are uncorrelated and ordered from PC1 onward according to the amount of variance they explain. The first n components explaining at least 90% of the total variance were retained for subsequent analyses.

For each DXA-derived variable, ordinary least squares (OLS) multiple linear regression models were constructed using anthropometric measures and CMJ principal components as predictors. The regression models were adjusted for age, body mass, height, waist circumference, and hip circumference. Body mass index was not included because it is a derived variable based on body mass and height and would therefore introduce redundancy with the selected anthropometric covariates. Model performance was evaluated through adjusted R^2^ [34], reflecting explained variance, alongside Akaike (AIC) and Bayesian (BIC) information criteria, log-likelihood, and CV-RMSE%, which was computed by scaling the root mean square error to the mean of the dependent variable to enable comparisons across differently scaled outcomes. Statistical significance was set at α = 0.05 (two-tailed). All analyses were conducted in Python 3.13.5 (Python Software Foundation) using NumPy 2.1.3, SciPy 1.15.3, Pandas 2.2.3, and statsmodels 0.14.4.

## 3. Results

The analysis included 110 postmenopausal women. 10 participants were excluded from the final analyses because no technically valid countermovement jump trial was available after quality control. Specifically, trials were excluded because of acquisition errors (e.g., loss of foot contact with the force platform or double bounces during the countermovement phase). Participants presented a mean body mass of 67.9 ± 14.1 kg and a height of 161.1 ± 5.1 cm. Mean waist and hip circumferences were 84.3 ± 12.3 cm and 103.8 ± 11.3 cm, respectively. The average age of the sample was 56.9 ± 3.8 years (range 46–68) and the time from menopause was 5.3 ± 2.9 years (range 1–10). Group means and standard deviations for CMJ metrics parameters are reported in Table 2.

### 3.1. Within-Session Reliability

Within-session reliability of the 17 CMJ-derived variables was assessed and the complete results are reported in Table A2. The ICCs ranged from 0.52 to 0.84, CVs from 1.1% to 30.1%, and SEM values from 0.07 to 140.51, depending on the measurement unit. Braking rate of force development showed moderate reliability compared with the more stable force-, impulse-, and temporal-domain variables, with an ICC of 0.56, CV of 30.1%, and SEM of 140.51 N·s^−1^. We have therefore avoided characterizing the reliability of this variable as excellent and have added a cautious interpretation of RFD-related findings.

### 3.2. Principal Component Analysis

**Figure 2:**
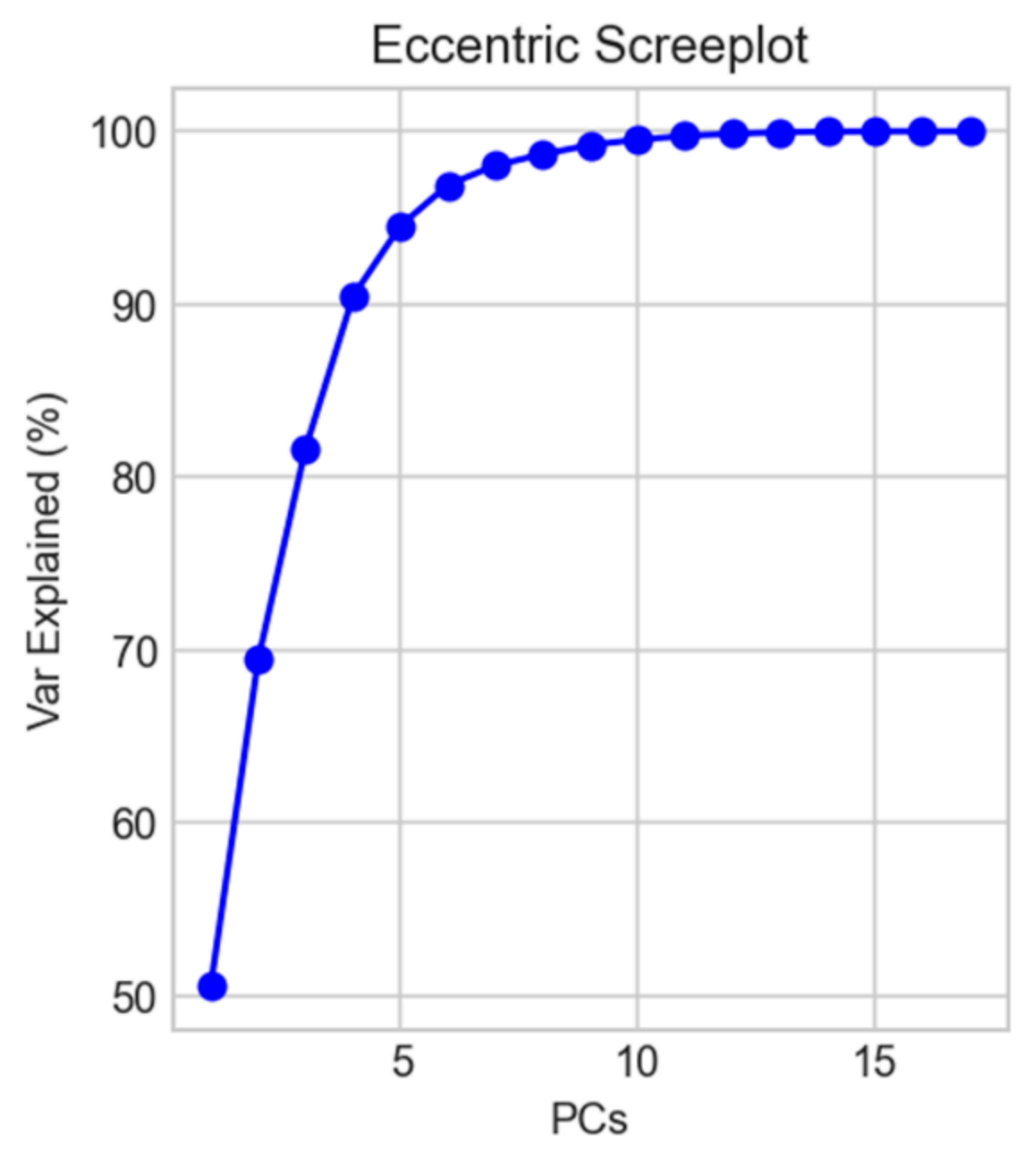
Scree plot of CMJ PCA for the variable set, illustrating the cumulative percentage of explained variance as a function of the number of principal components (PCs).

The PCA retained four principal components, which together accounted for 90.5% of the total variance in the 17 CMJ-derived variables (Figure 2).

**Figure 3:**
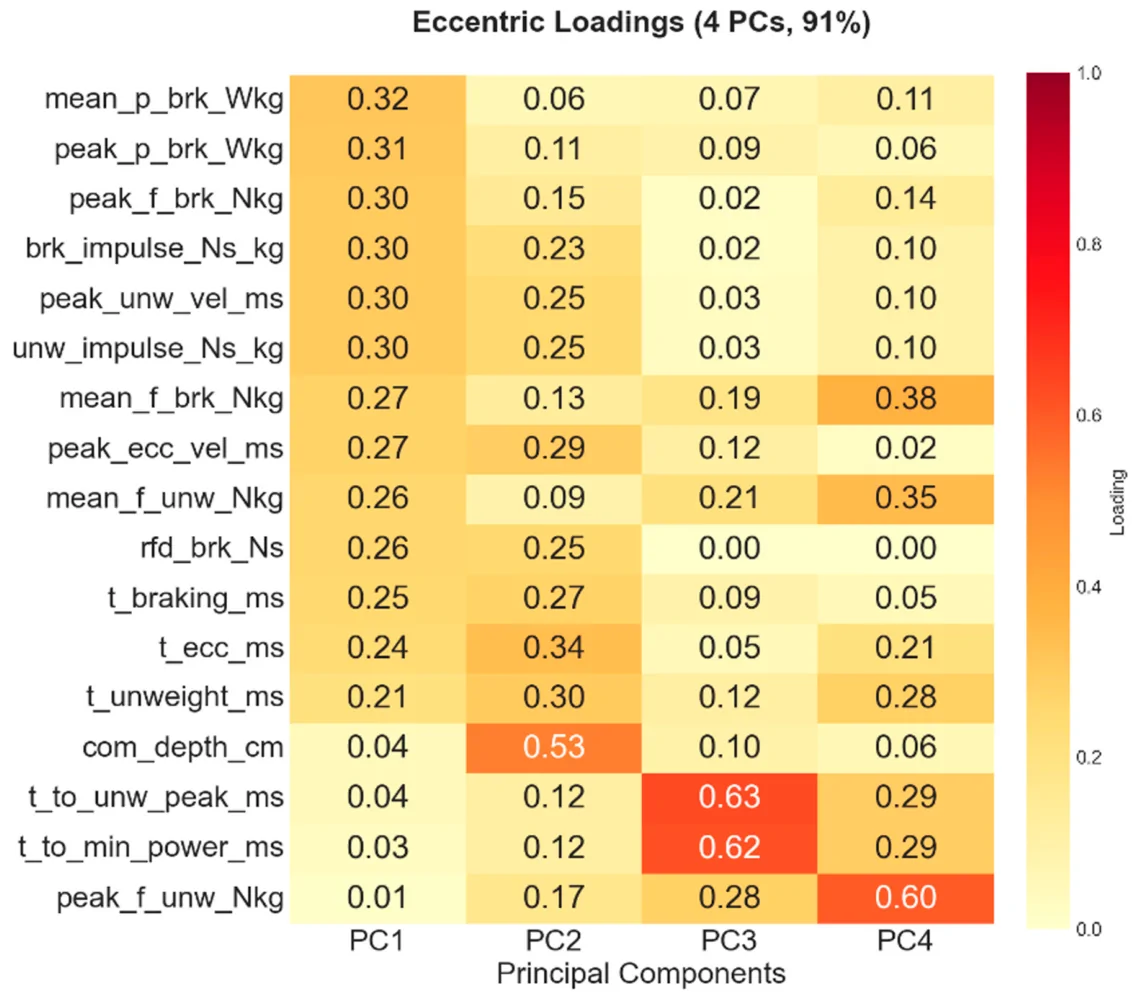
Heatmap representations of the loadings for each selected PCs. The color intensity (warmer tones for higher absolute loadings) highlights the variables contributing most strongly to each PC, facilitating biomechanical interpretation.

The loading coefficients for the four retained components are presented in Figure 3.

PC1 showed its largest absolute loadings for braking mean and peak force, braking peak and mean power, braking impulse, peak unloading descent velocity, and unloading impulse.

PC2 showed its largest absolute loadings for countermovement depth and the temporal variables describing the unloading and eccentric phases.

PC3 showed its largest absolute loadings for time to peak unloading force and time to minimum power.

PC4 showed its largest absolute loading for peak force during the unloading phase.

Heatmap representation of the loading coefficients for the four retained principal components. Greater color intensity denotes larger absolute loading coefficients.

### 3.3. Multiple Linear Regression Models

A comprehensive summary of model fit indices for all DXA parameters, including statistical significance and performance metrics, is provided in Table A1. The results indicate that the models were able to explain a substantial proportion of the variability in bone density and mass variables, with adjusted determination coefficients ($R_{\mathrm{adj}}^{2}$) typically ranging from 0.11 to 0.41 and CV-RMSE values between about 7% and 31%. The highest adjusted $R^{2}$ values were observed for trabecular BMC at the femoral neck $\left(R_{adj}^{2}=0.41\right)$, BMC at the femoral shaft $\left(R_{adj}^{2}=0.40\right)$, and total trabecular volume $\left(R_{adj}^{2}=0.39\right)$.

The complete AIC, BIC, log-likelihood, and CV-RMSE values for each outcome-specific model are reported in Table A1.

Among the four principal components, PC2 had the lowest *p*-value in most of the outcome-specific models, whereas PC1, PC3, or PC4 had the lowest *p*-value for selected outcomes. The overall F-tests were statistically significant for all of the models. None of the individual principal-component coefficients reached the prespecified statistical significance threshold of *p* < 0.05.

## 4. Discussion

The aim of the study was to determine whether distinct biomechanical profiles of the eccentric phase of the SSC during the CMJ were associated with DXA- and 3D-DXA-derived proximal femur properties in physically inactive women within the first 10 years after menopause. The findings suggest that multivariable models incorporating eccentric-phase mechanical profiles were associated with proximal femur DXA- and 3D-DXA-derived bone properties. Four principal components explained 90.5% of the variance in the biomechanical variables. Among these, PC2, characterized primarily by greater countermovement depth and longer eccentric duration, consistently showed the lowest *p*-values across most regression models, whereas PC1, characterized by higher eccentric force, power, and impulse, showed the lowest *p*-values for several trabecular outcomes. However, none of the individual principal components reached statistical significance. Taken together, these findings suggest that the overall eccentric mechanical profile, rather than any isolated biomechanical characteristic, may be associated with proximal femur bone properties in this population.

From a physiological perspective, the observed associations are consistent with Frost’s mechanostat theory and subsequent refinements in bone mechanobiology, which propose that bone adaptation depends on multiple loading characteristics, including strain magnitude, rate, frequency, and spatial distribution [8,10,35]. Accordingly, different eccentric loading profiles may represent distinct mechanical loading patterns whose biological relevance warrants further investigation in longitudinal and intervention studies.

A deeper countermovement combined with a longer eccentric phase (PC2) may increase time under tension and prolongs the duration of mechanical loading. Within the mechanostat framework, sustained strain exposure above the minimum effective threshold may modify the cumulative mechanical stimulus experienced by bone [9,36]. This type of loading is also associated with more homogeneous strain distribution across cortical and trabecular compartments, although whether this translates into measurable skeletal adaptations remains to be established experimentally [11]. Experimental and computational studies support that distributed, lower-rate loading promotes more uniform tissue-level strain fields, enhancing trabecular maintenance and cortical modelling [10,11]. From a neuromuscular standpoint, longer eccentric phases require sustained motor unit recruitment and controlled force transmission, which may also influence to consistent skeletal loading patterns, particularly relevant in aging populations with compromised bone properties [37].

In contrast, higher eccentric force, power, and impulse (PC1) represent loading profiles characterized by rapid force development and high strain rates. Bone tissue is highly sensitive to these dynamic loading conditions, as high strain rates have been shown to be associated with greater mechano-transductive signaling even when loading duration is short [8,10]. Explosive eccentric actions, supported by stretch-reflex mechanisms and elastic energy storage within the muscle–tendon unit, generate high peak forces and rapid deformation gradients [38]. These conditions have been associated with trabecular remodeling and volumetric adaptations, particularly in regions exposed to multidirectional loading. This mechanism may provide one possible explanation for the observed statistical association with trabecular volume. Additionally, variability in strain patterns induced by rapid loading may help prevent cellular desensitization, further enhancing osteogenic potential [8]. Overall, these mechanobiological considerations provide plausible explanations for the observed associations but should be interpreted as hypothesis-generating rather than as evidence of specific osteogenic mechanisms.

From a biomechanical assessment perspective, these exploratory findings may inform future research on exercise prescription for physically inactive women in early postmenopause; however, they do not establish that the identified mechanical characteristics are applicable to other postmenopausal populations. Future longitudinal and intervention studies should determine whether modifying eccentric movement strategies can influence skeletal adaptation. A substantial body of evidence supports the effectiveness of resistance training in postmenopausal women, with meta-analyses demonstrating improvements in BMD at clinically relevant sites such as the hip and lumbar spine [39,40]. More recent syntheses indicate that moderate-to-high intensity loading (≥70–85% 1RM), combined with controlled movement tempos, is particularly effective for promoting bone strength and structural adaptations [41,42]. Current exercise guidelines for osteoporosis prevention similarly recommend multi-joint, weight-bearing exercises performed with sufficient intensity and controlled execution to maximize osteogenic stimulus while ensuring safety [43].

Importantly, the CMJ was used in the present study as a standardized biomechanical assessment rather than as an exercise intervention. Therefore, the observed associations should not be interpreted as evidence that performing CMJs, or modifying eccentric movement strategies during CMJ execution, improves bone health. Instead, the present findings provide hypotheses that should be tested in longitudinal and randomized intervention studies designed to determine whether specific eccentric loading profiles can induce favorable skeletal adaptations.

This study presents several limitations that should be considered when interpreting the findings. First, its cross-sectional design precludes any causal inference regarding the relationship between eccentric CMJ mechanics and bone properties; thus, it cannot be determined whether specific loading strategies influence skeletal adaptation or whether individuals with more favorable bone properties preferentially adopt particular movement patterns. Consequently, the observed associations should not be interpreted as evidence that modifying CMJ mechanics would improve bone health. Second, although principal component analysis allowed meaningful dimensionality reduction, the resulting components represent composite constructs that may limit direct biomechanical interpretability and obscure the contribution of individual variables. In addition, participants performed the CMJ using a self-selected countermovement depth. Although this approach preserves natural movement strategy and is consistent with standardized maximal CMJ assessment protocols, it introduces inter-individual variability in movement execution that may influence the force–time characteristics included in the principal component analysis. Consequently, part of the variability captured by the identified biomechanical profiles may reflect differences in preferred movement strategy rather than differences in neuromuscular capacity alone. Future studies should investigate whether standardizing countermovement depth, or explicitly accounting for its influence, modifies the observed associations with bone properties. Third, internal bone loading was inferred from external force–time characteristics without direct quantification of joint contact forces or muscle forces through musculoskeletal modelling, which may lead to an incomplete representation of the mechanical stimuli experienced by bone.

Additionally, the study population was restricted to physically inactive women within the first 10 years after menopause who met the predefined eligibility criteria. Consequently, the findings should not be generalized to women at later postmenopausal stages, physically active or athletic women, women receiving hormone replacement therapy, or women with clinical characteristics not represented in the present cohort. Although women with obesity were included, the regression models accounted for body size and body shape by adjusting for body mass, height, waist circumference, and hip circumference, thereby reducing the likelihood that the observed associations were explained solely by anthropometric differences. Nevertheless, residual confounding related to body composition and other unmeasured factors cannot be completely excluded. Time since menopause, habitual physical activity, hormonal treatment, and overall health status may influence both CMJ mechanics and bone properties. Replication in larger and more clinically diverse postmenopausal populations is therefore warranted. Finally, although the multivariable models explained a meaningful proportion of the variability in several bone outcomes, none of the individual principal components reached statistical significance. Therefore, the present findings should be interpreted as exploratory and hypothesis-generating, suggesting that the overall eccentric mechanical profile, rather than any isolated biomechanical characteristic, may be associated with DXA- and 3D-DXA-derived bone properties. Confirmation in longitudinal and randomized intervention studies is required before any causal or clinical inferences can be made.

## 5. Conclusions

In conclusion, the present study indicates that multivariable models incorporating eccentric-phase mechanical profiles during the CMJ explain a meaningful proportion of the variability in proximal femur DXA- and 3D-DXA-derived bone properties in physically inactive women within the first 10 years after menopause. Although none of the individual principal components independently predicted the assessed bone outcomes, the findings suggest that the combined mechanical characteristics of the eccentric phase, rather than any isolated biomechanical variable, may be relevant to skeletal properties in this population. These results support the concept that internal loading characteristics, beyond external impact measures alone, may contribute to the mechanical stimuli associated with bone health. Future longitudinal and intervention studies are warranted to determine whether specific eccentric loading strategies can induce favorable skeletal adaptations in physically inactive women during early postmenopause.

## Figures and Tables

**Table 1:** A complete list of countermovement jump-derived variables calculated in this study, along with their respective units.

Variable Type	Variable	Unit
Kinematic variables	Countermovement center of mass depth (CoM displacement)	cm
	Unloading phase duration	ms
	Braking phase duration	ms
	Eccentric phase duration	ms
	Time to peak unloading force	ms
	Time to minimum power	ms
	Peak unloading descent velocity	m/s
	Peak eccentric velocity (negative)	m/s
Kinetic variables	Unloading peak force normalized	N/kg
	Unloading mean force normalized	N/kg
	Braking peak force normalized	N/kg
	Braking mean force normalized	N/kg
	Braking rate of force development	N/s
	Braking peak power normalized	W/kg
	Braking mean power normalized	W/kg
	Unloading impulse normalized	Ns/kg
	Braking impulse normalized	Ns/kg

CoM = center of mass; cm = centimeters; ms = milliseconds; m/s = meters per second; N/kg = Newtons per kilogram; N/s = Newtons per second; W/kg = watts per kilogram; Ns/kg = Newton-seconds per kilogram.

**Table 2:** Descriptive statistics for the CMJ-derived variables, expressed as means and standard deviations.

Variable Type	Variable Name	Unit	Mean (SD)
Kinematic variables	Countermovement center of mass depth (CoM displacement)	cm	19.11 (6.79)
	Unloading phase duration	ms	393 (123)
	Braking phase duration	ms	349 (144)
	Eccentric phase duration	ms	585 (178)
	Time to peak unloading force	ms	180 (70)
	Time to minimum power	ms	531 (141)
	Peak unloading descent velocity	m/s	0.36 (0.13)
	Peak eccentric velocity (negative)	m/s	0.66 (0.2)
Kinetic variables	Unloading peak force normalized	N/kg	9.37 (0.39)
	Unloading mean force normalized	N/kg	7.80 (0.86)
	Braking peak force normalized	N/kg	16.72 (3.35)
	Braking mean force normalized	N/kg	10.66 (0.84)
	Braking rate of force development	N/s	2681 (211)
	Braking peak power normalized	W/kg	1.77 (1.3)
	Braking mean power normalized	W/kg	1.26 (0.35)
	Unloading impulse normalized	Ns/kg	0.36 (0.13)
	Braking impulse normalized	Ns/kg	0.35 (0.13)

Abbreviations: SD = standard deviation.

## Data Availability

The data presented in this study are available from the corresponding author upon reasonable request. The data are not publicly available due to ethical and privacy restrictions concerning participant information.

## Abbreviations

The following abbreviations are used in this manuscript:

AIC	Akaike Information Criterion
BIC	Bayesian Information Criterion
BMC	Bone Mineral Content
BMD	Bone Mineral Density
BW	Body Weight
CMJ	Countermovement Jump
COM	Centre of Mass
CV-RMSE	Cross-Validated Root Mean Square Error
DXA	Dual-Energy X-ray Absorptiometry
HR-pQCT	High-Resolution Peripheral Quantitative Computed Tomography
OLS	Ordinary Least Squares
PCA	Principal Component Analysis
PC	Principal Component
SSC	Stretch-Shortening Cycle
vBMD	Volumetric Bone Mineral Density
vGRF	Vertical Ground Reaction Force

## Appendix A

**Table A1:** Model fit comparison for DXA parameters. Columns report F *p*-value of the model, R^2^, AIC, BIC, logLik, MSE residuals, best PC (lowest *p*-value among PCs) and the related *p*-value.

Variable	F *p*-Value	R^2^	AIC	BIC	logLik	CV-RMSE (%)	Best PC	*p*-Value Best PC
BMD Neck (g/cm^2^)	0.001	0.16	−158.74	−131.73	89.37	13	PC2	0.148
BMD Troch (g/cm^2^)	<0.001	0.28	−180.40	−153.39	100.20	14	PC2	0.100
BMD Shaft (g/cm^2^)	<0.001	0.28	−112.27	−85.26	66.13	12	PC2	0.136
BMD Total (g/cm^2^)	<0.001	0.29	−161.12	−134.12	90.56	12	PC2	0.111
BMC Neck (g)	<0.001	0.31	189.40	216.40	−84.70	13	PC2	0.129
BMC Troch (g)	<0.001	0.21	463.02	490.02	−221.51	20	PC2	0.083
BMC Shaft (g)	<0.001	0.40	448.79	475.79	−214.39	11	PC2	0.054
BMC Total (g)	<0.001	0.35	618.47	645.47	−299.23	13	PC2	0.050
Area Neck (cm^2^)	<0.001	0.22	84.33	111.34	−32.17	7	PC3	0.748
Area Troch (cm^2^)	0.015	0.11	416.32	443.33	−198.16	12	PC2	0.235
Area Shaft (cm^2^)	<0.001	0.30	230.64	257.64	−105.32	5	PC4	0.050
Area Total (cm^2^)	<0.001	0.31	449.70	476.70	−214.85	6	PC2	0.201
Trabecular vBMD Total (mg/cm^3^)	<0.001	0.27	1119.82	1146.82	−549.91	24	PC2	0.111
Trabecular BMC Total (g)	<0.001	0.38	439.27	466.27	−209.63	21	PC2	0.116
Trabecular BMC Neck (g)	<0.001	0.41	90.20	117.20	−35.10	18	PC2	0.255
Trabecular BMC Troch (g)	<0.001	0.35	236.87	263.88	−108.44	23	PC2	0.186
Trabecular BMC Shaft (g)	<0.001	0.36	255.57	282.57	−117.78	21	PC2	0.061
Integral vBMD Neck (mg/cm^3^)	0.012	0.11	1211.57	1238.57	−595.78	16	PC2	0.155
Integral vBMD Troch (mg/cm^3^)	<0.001	0.31	1133.50	1160.51	−556.75	17	PC2	0.136
Integral vBMD Shaft (mg/cm^3^)	<0.001	0.24	1225.28	1252.28	−602.64	16	PC2	0.059
Trabecular vBMD Neck (mg/cm^3^)	<0.001	0.17	1156.85	1183.85	−568.42	20	PC2	0.149
Trabecular vBMD Troch (mg/cm^3^)	<0.001	0.27	1076.30	1103.30	−528.15	23	PC2	0.173
Trabecular vBMD Shaft (mg/cm^3^)	<0.001	0.22	1137.62	1164.62	−558.81	25	PC2	0.079
Trabecular Volume Total (cm^3^)	<0.001	0.39	710.40	737.40	−345.20	11	PC1	0.467
Trabecular Volume Neck (cm^3^)	<0.001	0.35	346.45	373.45	−163.22	12	PC4	0.264
Trabecular Volume Troch (cm^3^)	<0.001	0.36	529.78	556.79	−254.89	11	PC1	0.366
Trabecular Volume Shaft (cm^3^)	<0.001	0.34	535.88	562.88	−257.94	12	PC3	0.221

**Table A2:** Within-session reliability of the 17 variables extracted from the CMJ tests.

Variable Type	Variable Name	ICC (95% CI)	CV (%)	SEM
Kinematic variables	Countermovement center of mass depth (CoM displacement)	0.68 (0.55–0.77)	17.7	3.9
	Unloading phase duration	0.52 (0.37–0.65)	17.9	38.1
	Braking phase duration	0.64 (0.52–0.74)	17.7	50.7
	Eccentric phase duration	0.60 (0.46–0.72)	15.5	17.2
	Time to peak unloading force	0.69 (0.58–0.77)	16.2	0.9
	Time to minimum power	0.69 (0.57–0.77)	15.9	0.9
	Peak unloading descent velocity	0.70 (0.59–0.78)	19.5	0.1
	Peak eccentric velocity (negative)	0.76 (0.67–0.83)	15.2	0.1
Kinetic variables	Unloading peak force normalized	0.55 (0.05–0.60)	1.1	0.3
	Unloading mean force normalized	0.63 (0.50–0.73)	5.6	0.5
	Braking peak force normalized	0.84 (0.78–0.89)	6.9	1.2
	Braking mean force normalized	0.58 (0.44–0.69)	3.5	0.5
	Braking rate of force development	0.56 (0.53–0.61)	30.1	140.5
	Braking peak power normalized	0.74 (0.64–0.81)	26.6	0.7
	Braking mean power normalized	0.75 (0.65–0.82)	24.5	0.1
	Unloading impulse normalized	0.70 (0.59–0.78)	19.5	0.1
	Braking impulse normalized	0.69 (0.57–0.77)	20.8	0.1
